# Dosimetric advantages of dual arc increments for head and neck volumetric‐modulated arc therapy in the Monaco treatment planning system

**DOI:** 10.1002/acm2.14571

**Published:** 2024-11-29

**Authors:** Jin Hwa Choi, Hyejo Ryu, Do Hoon Oh, Lee Yoo, Minsoo Chun

**Affiliations:** ^1^ Department of Radiation Oncology Chung‐Ang University College of Medicine Seoul Republic of Korea; ^2^ Department of Radiation Oncology Chung‐Ang University Gwang Myeong Hospital Gyeonggi‐do Republic of Korea; ^3^ Institute of Radiation Medicine Seoul National University Medical Research Center Seoul Republic of Korea

**Keywords:** gantry arc increment, head and neck radiotherapy, radiotherapy planning, volumetric‐modulated arc therapy

## Abstract

**Purpose:**

This study aimed to assess the dosimetric advantages of dual arc increments in head and neck volumetric‐modulated arc therapy (VMAT) in the Monaco treatment planning system (TPS).

**Methods:**

Three VMAT plans were created for each of the 10 patients by prescribing 70 Gy in 35 fractions with arc increment combinations of 30°/30°, 15°/15°, and 30°/15° in the Monaco TPS. The dose to 0.03 cm^3^ volume (D_0.03cc_), conformity number, and homogeneity and gradient indices was compared for the planning target volume (PTV), and the D_0.03cc_ and mean dose (D_mean_) of the spinal cord, brain stem, parotid glands were compared. For plan complexity evaluation, the monitor unit and various related metrics were compared. Wilcoxon signed‐rank tests were performed across plans for the evaluated indicators.

**Results:**

For PTV, plans with 30°/15° showed comparable D_0.03cc_ and homogeneity and gradient indices to those of plans with 30°/30° and 15°/15° while exhibiting a better conformity number. The D_0.03cc_ for spinal cord and brain stem for plans with 30°/15° were 26.0% and 20.8% less than those with 30°/30° and 16.8% and 19.0% less than those with 15°/15°, respectively. The D_mean_ for the left and right parotid glands under plans with 30°/15° were 17.4% and 13.2% less than those with 30°/30° and 14.0% and 9.8% less than those with 15°/15°, respectively. The total monitor unit in plans with 30°/15° was less than that in other plans but with no significance. The plans with 30°/15° showed higher modulation complexity and plan‐averaged irregularity, while no significant differences observed in both plan‐averaged area and modulation compared with other plans.

**Conclusion:**

In head and neck VMAT, a dual arc increment of 30°/15° seems advisable because it can substantially reduce doses to normal tissues with comparable delivery efficiency while maintaining target dose coverage.

## 1 INTRODUCTION

As volumetric‐modulated arc therapy (VMAT) can maximize tumor control probability while reducing the probability of normal tissue complications, it is widely used in radiotherapy.^1^, ^2^, ^3^ VMAT relies on numerous mechanical parameters and treatment planning techniques.^3^ For instance, this technique largely depends on the types of multi‐leaf collimators (MLCs) and linear accelerators as well as optimization algorithms implemented in treatment planning systems (TPS).

At our clinic, we use a VersaHD linear accelerator (Elekta AB, Stockholm, Sweden) paired with the Monaco 6.1.2 TPS (Elekta AB). The VMAT optimization algorithm in the Monaco TPS incorporates a constrained optimization technique called the Hyperion VMAT sequencer (Elekta AB).^4^ The sequencer characterizes MLC sequences with correlated leaf movements and synchronizes leaf direction changes. In the first stage of VMAT optimization, the gantry rotation angle is split into arc sectors, and each sector is associated with a fixed angle, where a fluence profile is formulated as fixed‐gantry intensity modulation radiation therapy optimization. The number of arc sectors may be determined by dividing gantry rotation angles by arc increment. The MLC changes its movement direction at each sector boundary; thus, it moves in a direction in a sector and the opposite direction in the next sector. The number of sectors is the same as the number of leaf direction changes minus one. A large number of sectors (i.e., small arc increments) may enable high levels of modulation while increasing the delivery time. By contrast, using a few sectors may accelerate delivery but with low levels of modulation, possibly resulting in low‐quality plans.^4^ Therefore, the selection of the optimal gantry arc increment is crucial in plan quality and delivery time. Elekta recommends a 30° arc increment for general VMAT plans, as this provides the best modulation efficiency by focusing on achieving a homogeneous dose distribution.^5^ This increment could be reduced if the plan requires more severe modulation. Nevertheless, few studies have addressed the effect of arc increment on plan quality. Chen et al. assessed the influence of arc increment on plan quality for cervical cancer patients, demonstrating that a 30° arc increment was the optimal setting for high plan quality and delivery efficiency.^6^ Nithya et al. recommended a large arc increment in VMAT for esophageal cancer by showing superior dose coverage and homogeneity index (HI) with a reduced monitor unit (MU).^7^

We hypothesized that the use of two different arc increments increases the degrees of freedom of the MLC, thereby improving plan quality while maintaining the level of modulation. To date, the effects of dual arc increment combinations in full‐arc VMAT optimization have not been extensively studied, particularly in the context of head and neck treatments, where the anatomy is complex and radiosensitivity is high. Given the intricate nature of head and neck anatomy and the importance of sparing radiosensitive organs, VMAT is particularly effective at delivering precise doses while protecting adjacent organs at risk (OARs). In this study, we present findings from three types of dual arc head and neck VMAT plans, utilizing 30°/30°, 15°/15°, and 30°/15° arc increment combinations. Our aim is to demonstrate the dosimetric advantages of these dual arc configurations in terms of dose‐volume metrics and the overall complexity of VMAT plans for head and neck treatments.

## 2 METHODS

### 2.1 Data selection and treatment planning

Ten patients were retrospectively selected following institutional review board approval and received treatment over 7 weeks in 35 fractions. The characteristics of the target and OAR are shown in Table 1. All the patients underwent computed tomography (CT) scans with a Big Bore RT CT simulator (Philips Health Systems, Cleveland, Ohio, USA), slice thickness of 2 mm, and appropriate immobilization using devices such as the iBEAM Overlay Adapter (CQ Medical, Avondale, Pennsylvania, USA) with Type‐S intensity modulation radiation therapy Reinforced Style Mask (CQ Medical, Avondale, PA) and a Precise Bite Patient Re‐Positioner (CQ Medical). OARs were automatically delineated by using the Contour ProtégéAI module of MIM Software (version 7.2.10; Cleveland, OH) and confirmed by two radiation oncologists. The clinical target volume was delineated according to relevant guidelines,^8^, ^9^, ^10^ and the planning target volume (PTV) was obtained by expanding the clinical target volume with a 3 mm margin. The CT images and structure sets were then transferred to the Monaco TPS in the DICOM format.

**TABLE 1:** Detailed characteristics of target and OARs for each patient.

Patient #	Treatment site	Volume (cc)					Distance to PTV (center‐to‐center, cm)			
		Target	Spinal cord	Brain stem	PG_L	PG_R	Spinal cord	brain stem	PG_L	PG_R
1	Hypopharynx & Neck	697.19	40.56	23.91	14.50	12.87	5.64	13.88	11.96	9.94
2	Hypopharynx	418.36	28.50	26.28	24.01	23.86	5.33	13.59	10.26	10.53
3	Nasopharynx	771.38	30.38	24.69	43.90	38.94	8.38	10.29	8.54	8.35
4	Nasopharynx	578.28	29.76	24.6	26.21	23.85	6.88	10.55	8.19	8.65
5	Oropharynx & Neck	541.35	20.10	24.17	25.45	24.54	7.26	9.79	6.95	7.87
6	Hypopharynx & Neck	691.96	38.66	25.01	18.20	17.86	7.04	13.09	11.09	9.65
7	Nasopharynx & Neck	706.52	42.03	31.17	50.85	53.08	9.36	10.08	8.77	8.78
8	Larynx & Neck	373.81	21.82	24.98	43.07	37.00	5.72	10.80	8.10	8.42
9	Larynx & Neck	323.48	31.52	30.30	25.69	28.51	6.65	12.51	9.84	10.04
10	Hypopharynx & Neck	716.45	30.68	23.89	22.13	27.03	5.82	12.10	10.11	9.16

Abbreviations: *PG_L*, Left parotid gland; *PG_R*, Right parotid gland.

**FIGURE 1:**
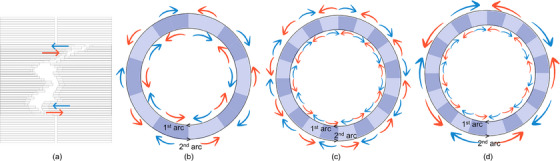
Schematic of the study concept. (a) Directions of MLC movement (color arrows) and arc increment combinations of (b) 30°/30° (group A), (c) 15°/15° (group B), and (d) 30°/15° (group C).

VMAT planning was established with two full arcs using 6 MV photon beams in the Monaco TPS (Version 6.1.2, Elekta AB). Monaco TPS allows users to specify arc increments between 5° and 45°.^5^ However, increments less than or equal to 10° or greater than or equal to 35° have been shown to be inappropriate to meet the requirements of dose‐volumetric parameters.^5^ Therefore, in this study, we narrowed the increment range to be between 15° and 30°. To examine the effects of different arc increment combinations, we created three plans for each patient using the following combinations: 30°/30° (group A), 15°/15° (group B), and 30°/15° (group C). A schematic of the study is shown in Figure 1. The collimator angles were set to 45°/315° per field to achieve the highest number of degrees of freedom for all plans,^11^, ^12^ and other parameters were fixed for a fair comparison.

The PTV was prescribed as 70 Gy in 35 fractions with the following OAR constraints: maximum dose (*D*_max_) for spinal cord less than 45 Gy, *D*_max_ for brain stem less than 54 Gy, and mean dose (*D*_mean_) for left and right parotid glands less than 26 Gy.^8^, ^9^, ^10^ The plans were optimized to achieve 100% of the prescription dose to cover 95% of the PTV and doses to 0.03 cm^3^ (*D*_0.03cc_) of the PTV less than 110% of the prescription dose. When these two conditions were not satisfied, the optimization parameters were modified until at least the PTV coverage was reached. Once optimization was completed, all the plans were normalized as 100% of the prescription dose to cover 95% of the PTV. To calculate the dose distributions, a Monte Carlo dose calculation with a statistical uncertainty of 1.0% per calculation and 2 mm grid resolution was used.^13^, ^14^, ^15^

### 2.2 Dose‐volumetric evaluation

In comparing dose‐volumetric parameters, *D*_0.03cc_ for the PTV, spinal cord, and brain stem and *D*_mean_ for the left and right parotid glands were compared for groups A–C. For a detailed evaluation of the PTV, the conformity number (CN), HI, and gradient index (GI) were investigated. CN was calculated as

(1) $$CN=\frac{V_{T,\mathrm{ref}}}{V_{T}}\;\times \frac{V_{T,\mathrm{ref}}}{V_{\mathrm{ref}}},$$

where *V_T_* is the volume of the PTV, *V*_ref_ is the total volume covered by the prescription dose, and *V*_T,ref_ is the volume of the PTV covered by the prescription dose.^16^ For plans with high conformity, CN approached 1. HI was calculated as

(2) $$HI=\frac{D_{2\%}-D_{98\%}}{D_{50\%}}\;,$$

where *D_x_*_%_ is the dose received by *x*% of the PTV.^17^ HI was close to zero for plans with highly homogeneous dose distributions. GI was calculated as

(3) $$GI=\frac{V_{50\%}}{V_{100\%}}\;,$$

where *V_y_*_%_ is the volume covered by *y*% of the prescription dose.^18^, ^19^ Plans with a steep dose gradient showed a GI close to 1.

### 2.3 Plan complexity calculation

To demonstrate the impact of different arc increment combinations on the level of plan modulation, various plan complexity metrics were calculated using MATLAB (MathWorks, Natick, Massachusetts, USA). We utilized the total MU, modulation complexity score for VMAT (MCS_v_), plan‐averaged area (PA), plan‐averaged irregularity (PI), and plan‐averaged modulation (PM), as suggested by Masi et al. and Du et al.^20^, ^21^ The calculation details are provided in Table 2. MCS_v_ approaches 0 for plans with high modulation, while an MCS_v_ value of 1 indicates no modulation.^20^ Higher PA, PI, and PM indicate large beam area, high irregularity, and high modulation, respectively.^21^

**TABLE 2:** Plan complexity metrics used in this study.

Metric	Control point phase	Arc phase	Plan phase	Reference
MCS_v_	$pos_{\max}\;\left(CP\right)={\left\langle \max\left(pos_{n\in N}\right)-\min\left(pos_{n\in N}\right)\right\rangle }_{\mathrm{leafbank}}$ $\;LSV_{\mathrm{cp}}={\left(\frac{\sum_{n=1}^{N-1}\left(pos_{\max}-\left|\left(pos_{n}-pos_{n+1}\right)\right|\right)}{\left(N-1\right)\times pos_{\max}}\right)}_{\mathrm{leftbank}}$ $\times {\left(\frac{\sum_{n=1}^{N-1}\left(pos_{\max}-\left|\left(pos_{n}-pos_{n+1}\right)\right|\right)}{\left(N-1\right)\times pos_{\max}}\right)}_{\mathrm{rightbank}}$ $\;AAV_{\mathrm{cp}}=\frac{\sum_{a=1}^{A}\left({\left\langle pos_{a}\right\rangle }_{\mathrm{leftbank}}-{\left\langle pos_{a}\right\rangle }_{\mathrm{rightbank}}\right)}{\sum_{a=1}^{A}\left({\left\langle \max(pos_{a})\right\rangle }_{\mathrm{leftbank}\in \mathrm{arc}}-{\left\langle \max(pos_{a})\right\rangle }_{\mathrm{rightbank}\in \mathrm{arc}}\right)}$	$\;MCS_{\mathrm{arc}}=\sum_{i=1}^{I-1}\left[\frac{AAV_{{\mathrm{cp}}_{i}}+AAV_{{\mathrm{cp}}_{i+1}}}{2}\right]\times \left[\frac{LSV_{{\mathrm{cp}}_{i}}+LSV_{{\mathrm{cp}}_{i+1}}}{2}\right]\times \frac{MU_{{\mathrm{cp}}_{i,i+1}}}{MU_{\mathrm{arc}}}$	$\;MCS_{v}=\frac{1}{K}\;\sum_{k=1}^{K}MCS_{{\mathrm{arc}}_{k}}$	Masi et al.^20^
PA	$\;AA_{ij}=\sum_{k=1}^{N_{LP}}t\cdot \left(x2_{ijk}-x1_{ijk}\right)$	$\;BA_{i}=\frac{\sum_{j}\left(MU_{ij}\cdot AA_{ij}\right)}{MU_{i}}$	$PA=\frac{\sum_{i}\left(BA_{i}\cdot MU_{i}\right)}{MU_{p}}$	Du et al.^21^
PI	$\;AI_{ij}=\frac{AP_{ij}^{2}}{4\pi \cdot AA_{ij}}$	$\;BI_{i}=\frac{\sum_{j}\left(MU_{ij}\cdot AI_{ij}\right)}{MU_{i}}$	$PI=\frac{\sum_{i}\left(BI_{i}\cdot MU_{i}\right)}{MU_{p}}$	Du et al.^21^
PM	–	$\;BM_{i}=1-\frac{\sum_{j}\left(MU_{ij}\cdot AA_{ij}\right)}{MU_{i}\cdot U\left(AA_{ij}\right)}$	$PM=\frac{\sum_{i}\left(BM_{i}\cdot MU_{i}\right)}{MU_{p}}$	Du et al.^21^

Abbreviations: *pos*, leaf position; *LSV*, leaf sequence variability; *AAV*, aperture area variability; *t*, width of leaf pair (fixed to 5 mm); *AA*, aperture area; *BA*, beam area; *AP*, aperture perimeter of MLC openings; *AI*, aperture irregularity; *BI*, beam irregularity; *BM*, beam modulation; U(*AA_ij_*), union area of all apertures per field.

### 2.4 Statistical analysis

Statistical analysis was performed for all dose‐volumetric parameters and plan complexity metrics. Because of the limited sample sizes in this study, the Wilcoxon signed‐rank test was applied to identify significant differences between groups.^22^ Data analysis was performed using the Real Statistics Resource Pack software (Release 9.1.1). Differences with *p*‐values less than 0.05 were considered significant.

## 3 RESULTS

### 3.1 Dose‐volumetric evaluation

**FIGURE 2:**
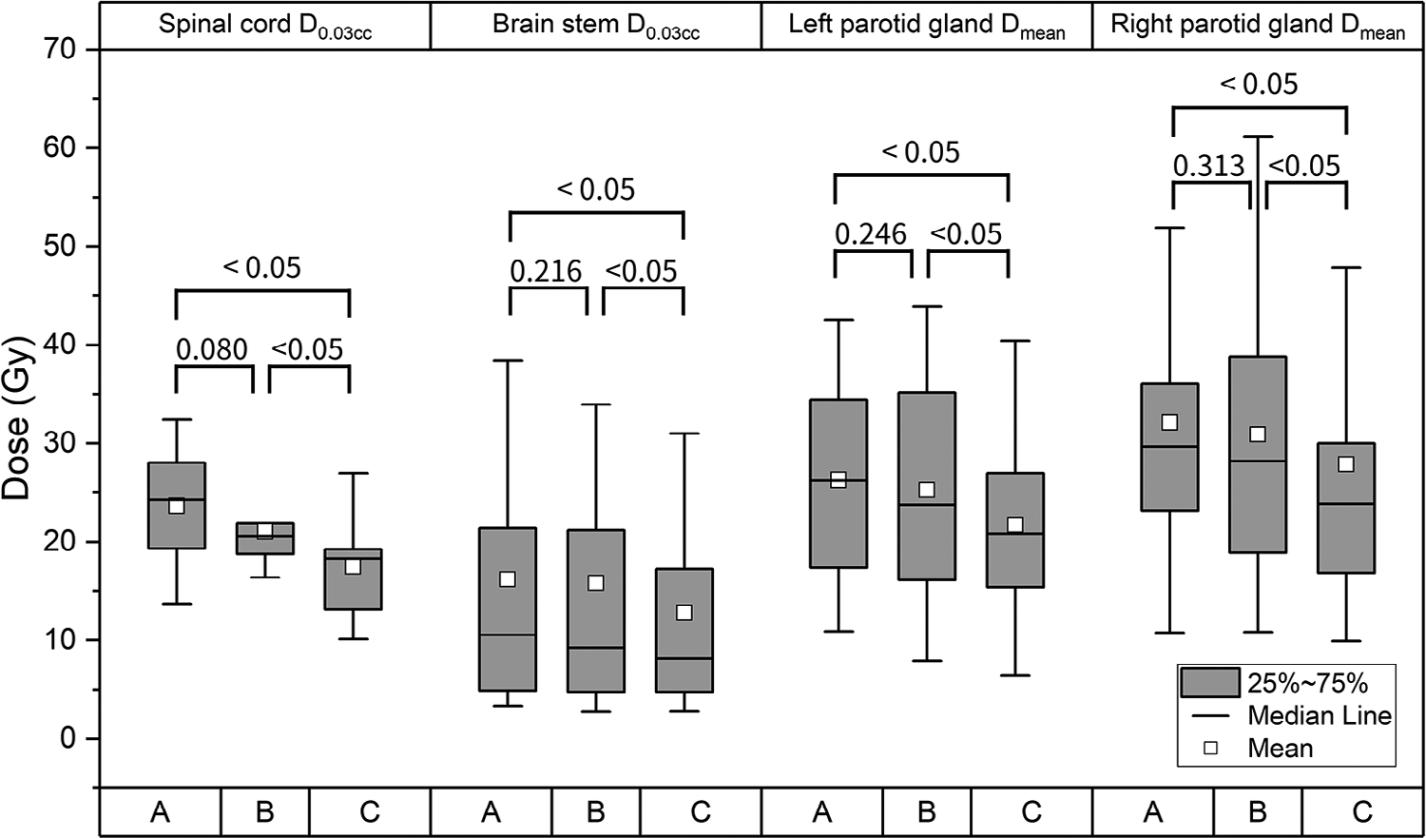
Box plots of *D*_0.03cc_ for the spinal cord and brain stem, and *D*_mean_ for parotid glands between groups. Numerical values above the parentheses indicate p‐values between groups calculated using the Wilcoxon signed‐rank test.

The statistics of the dose‐volumetric parameters per group are listed in Table 3. The *D*_0.03cc_ values for the spinal cord and brain stem in group C were 26.0% and 20.8% less than those in group A, and 16.8% and 19.0% less than those in group B, respectively. The *D*_mean_ values for the left and right parotid glands in group C were 17.4% and 13.2% less than those in group A, and 14.0% and 9.8% less than those in group B, respectively. *D*_0.03cc_ for the spinal cord and brain stem, and *D*_mean_ for parotid glands in group C had significant differences from those in the other groups, with all the *p*‐values being less than 0.05 (Figure 2). CN and HI for the PTV showed similar numerical values, while GI for group C had the steepest gradient, indicating the lowest GI with significance (*p *< 0.05).

**TABLE 3:** Mean and standard deviation of dose‐volumetric parameters and *p*‐values across groups.

	Mean ± standard deviation			*p*		
Dose‐volumetric parameter	A	B	C	A–B	A–C	B–C
PTV						
*D*_0.03cc_	7624.9 ± 74.6	7648 ± 103.19	7607.7 ± 44.12	0.097	0.246	0.216
CN	0.85 ± 0.04	0.86 ± 0.04	0.86 ± 0.04	0.019	0.003	0.348
HI	0.06 ± 0.01	0.06 ± 0.01	0.06 ± 0.01	0.246	0.313	0.348
GI	3.03 ± 0.56	2.93 ± 0.46	2.83 ± 0.48	0.097	0.002	0.042
Spinal cord						
*D*_0.03cc_	2365.1 ± 591.9	2104.4 ± 504.1	1750.2 ± 489.9	0.080	0.001	0.002
*D*_mean_	1406.7 ± 441.8	1296.0 ± 386.9	1086.6 ± 405.7	0.161	0.001	0.001
Brain stem						
*D*_0.03cc_	1618.4 ± 1633.0	1581.1 ± 1676.1	1281.1 ± 1185.6	0.216	0.002	0.005
*D*_mean_	626.1 ± 782.2	658.5 ± 864.7	520.4 ± 588.2	0.246	0.007	0.001
Left parotid gland						
*D*_0.03cc_	7143.8 ± 579.2	7162.4 ± 478.3	7128.6 ± 623.9	0.461	0.385	0.423
*D*_mean_	2629.1 ± 1070.9	2525.1 ± 1174.4	2170.9 ± 977.2	0.246	0.001	0.002
Right parotid gland						
*D*_0.03cc_	7122.1 ± 695.1	7084.9 ± 686.2	7102.9 ± 674.7	0.053	0.188	0.216
*D*_mean_	3211.7 ± 1472.8	3088.7 ± 1527.2	2787.2 ± 1464.1	0.313	0.001	0.002

**FIGURE 3:**
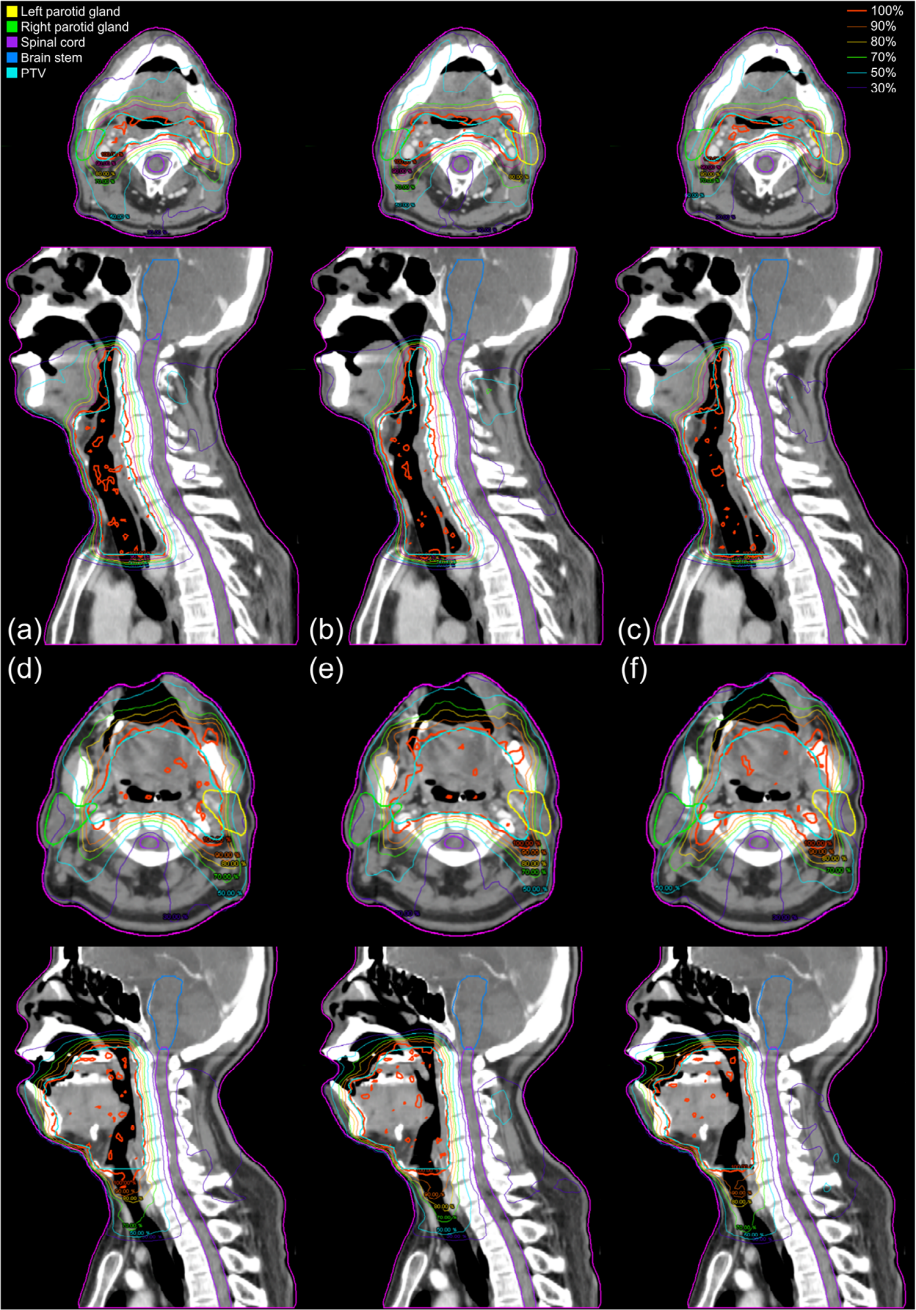
Dose distributions for two representative cases. One with a low level of PM for groups (a) A, (b) B, and (c) C, and the other with an elevated level of PM for groups (d) A, (e) B, and (f) C. Color information for the structure sets and isodose lines is presented in the upper‐left and upper‐right corners, respectively.

**FIGURE 4:**
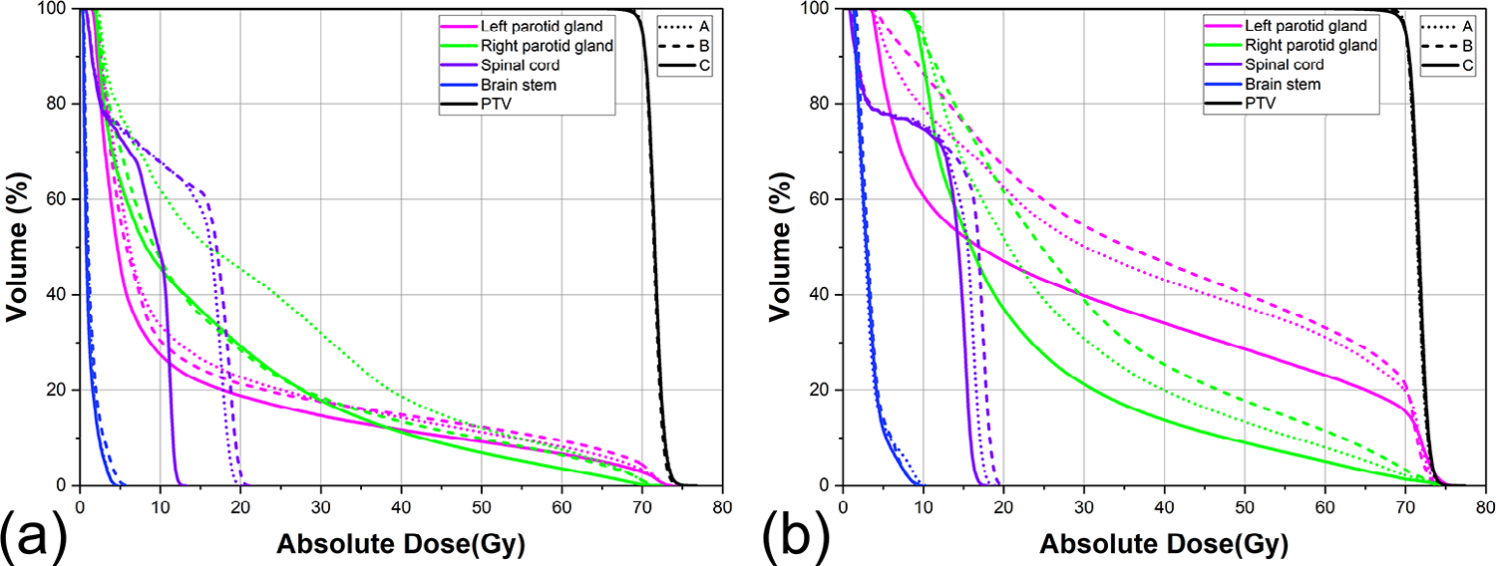
DVHs for two selected patients. One with a low level of PM (a), and the other with an elevated level of PM (b). Graphs are shown with dotted, dashed, and solid lines for arc increment combinations of 30°/30°, 15°/15°, and 30°/15°, respectively.

The dose distributions in axial slices and dose–volume histograms (DVHs) for the patients are shown in Figures 3 and 4. The 30% isodose lines (purple) in the spinal cord and parotid glands for group C were much narrower than those for groups A and B. The DVHs also presented a more notable dose reduction for OARs in group C than in groups A and B, while similar dose coverages were observed for the PTV.

### 3.2 Plan complexity calculation

The mean and standard deviation of the plan complexity metrics are listed in Table 4. The mean MCS_v_ for group B was the smallest but not significantly different from that for group C, indicating that group B had the highest level of plan modulation. The mean total MU in group C was the lowest but without significant differences with other groups. The mean PA in group A was the highest, and that in group B was the smallest. PI in group C was the highest among the groups. MCS_v_, PA, and PI between groups A and B showed significant differences, while all the metrics between groups B and C showed no significant differences.

**TABLE 4:** Mean and standard deviation of plan complexity metrics and *p*‐values across groups.

	Mean ± standard deviation			*p*		
Plan complexity metric	A	B	C	A–B	A–C	B–C
MCS_v_	0.17 ± 0.08	0.09 ± 0.01	0.1 ± 0.03	0.007	0.010	0.161
MU	1268.2 ± 225.9	1309.7 ± 223.2	1240.6 ± 157.2	0.216	0.313	0.138
PA	32.0 ± 6.2	29.9 ± 4.1	31.4 ± 8.2	0.042	0.385	0.138
PI	200.2 ± 67.2	265.13 ± 112.0	284.2 ± 66.5	0.005	0.001	0.385
PM	0.86 ± 0.02	0.87 ± 0.02	0.87 ± 0.02	0.065	0.461	0.188

## 4 DISCUSSION

In this study, we demonstrated that the combination of 30°/15° arc increments reduced both *D*_0.03cc_ for the spinal cord and brain stem and *D*_mean_ for the left and right parotid glands by 26.0%, 20.1%, 17.4%, and 13.2% compared with those of 30°/30° and by 16.8%, 19.0%, 14.0%, and 9.8% compared with those of 15°/15°, respectively. At the same time, *D*_0.03cc_, CN, HI, and GI for the PTV did not vary significantly. The mean total MU in group C was the lowest among the groups, but the differences were not significant. Plan complexity metrics such as MCS_v_, PA, PI, and PM between groups A and B showed significant differences. Thus, the arc increment combination of 15°/15° (group B) showed a high level of plan modulation and irregularity with smaller beam apertures, suggesting the potential improvement in plan quality with an enhanced level of modulation compared with the combination of 30°/30°.

In the statistical analysis between groups B and C, all the plan complexity metrics did not show any significant differences, indicating similar plan complexities for these groups. In the comparison between groups A and C, only MCS_v_ and PI showed noticeable differences, whereas the differences in MU, PA, and PM were not significant. The plan complexity metrics showed that groups B and C had similar levels of complexity, and fluctuations between groups A and C depended on the definition of plan complexity. As reducing the arc increment increased plan quality by inducing higher levels of modulation on MLCs, high plan complexity was expected in group B. However, the combinations of different arc increments in this study showed no significant differences with combination 15°/15° and did not show high complexity with combination 30°/30°.

Initially, we tried to reduce the dose to OARs based on relevant guidelines by considering *D*_max_ < 45 and 54 Gy for the spinal cord and brain stem, respectively, and *D*_mean_ < 26 Gy for both parotid glands. Various dose‐volumetric conditions were not satisfied in some patients depending on the inclusion or close location of OARs in the PTV, even under the arc increment combination of 30°/15°. Nonetheless, group C exhibited superior dose reduction compared with groups A and B. Thus, a radiation oncologist has various options to consider other clinical judgments.

In previous studies, trials have been conducted to demonstrate the impact of arc increment on VMAT. Nithya et al. demonstrated that the use of a larger gantry angle increment outperformed the use of a smaller angle in terms of dose coverage, HI, CI, and normal tissue sparing in VMAT for esophageal cancer patients.^7^ They set single arc increments of 15°, 20°, 30°, and 40° for comparison in terms of doses to targets and OARs. Although the doses to OARs with a 15° arc increment were the lowest, they concluded that plans with 40° increment were optimal because the total MUs in the 40° increment were comparable to those with 30° increment (control group). In other words, a smaller arc increment could increase the plan quality but failed to increase the delivery efficiency. Chen et al. found that the use of a 30° arc increment for VMAT in cervical cancer patients was optimal because dose‐volumetric parameters in OARs were lower than those for 20° and 40° arc increments, while the treatment time with a 30° arc increment was the shortest.^6^ However, dose reduction according to arc increment did not exhibit significant differences.

In this study, we confirmed that the use of dual arc increments (group C) reduced the average *D*_mean_ to approximately 22 and 28 Gy in the left and right parotid glands, respectively. Deasy et al. reported that severe xerostomia could be prevented in the low‐risk group if at least one parotid gland received a *D*_mean_ less than 20 Gy or both glands received a *D*_mean_ less than 25 Gy.^23^, ^24^, ^25^ In our study, six patients in group C were classified as low risk, and one of these patients would have been classified as high risk if a single‐arc technique (Groups A and B) had been used. Considering these findings, we speculated that the dual‐arc increment technique could reduce the probability of moderate‐to‐severe xerostomia after 12 months of treatment to less than 20%.^23^, ^24^, ^25^ Together, these findings suggest that regardless of the risk classification, the dual‐arc increment approach used in Group C significantly lowers doses to the parotid glands.

This study has some limitations. First, the sample size was relatively small. To address this, we used the Wilcoxon signed‐rank test, a robust non‐parametric analysis useful for small sample sizes and non‐normally distributed data, which compares the medians of two related groups. Despite the limited sample size, group C demonstrated significantly lower mean DV parameters than groups A and B, with no remarkable deviations in plan complexity metrics. We acknowledge that a larger sample size would enhance the statistical power and generalizability of our findings, and future studies with a larger cohort are necessary to validate these results and further explore the observed trends. Second, the choice of collimator angles may have affected the plan quality, as user‐defined parameters in VMAT planning can lead to variations in plan quality, with collimator angles being a critical factor affecting the outcome. We chose collimator angles of 45° and 315° for each field based on literature suggesting that a 90° difference between angles (*Δθ*) offers the most beneficial dosimetric effects.^12^ Although *Δθ *= 90° with various combinations, including 280°/10°, 300°/30°, 315°/45°, 330°/60°, and 350°/80° showed no significant differences in plan quality,^12^ minor dose differences were reported in the previous study; nevertheless, their effects on OAR sparing was negligible.^12^ Finally, rather than performing actual delivery with gamma analysis, we used plan complexity metrics to predict plan deliverability. Lower gamma passing rates in patient‐specific quality assurance, that is, low plan deliverability, are generally associated with plans with higher PM or lower MCS values.^20^, ^21^, ^26^, ^27^, ^28^ Because PA and PM did not exhibit significant differences across groups, the deliverability or gamma passing rates would be similar. MCS_v_ showed significant differences between groups A and B, with group B showing increased modulation. This could be because a small arc increment generates more sectors, leading to more severe MLC modulation during optimization. Although MCS_v_ showed no significant differences between groups A–C and B–C, the degree of modulation with the 15°/15° combination is expected to be greater than those with 30°/30° and 30°/15°. Although a larger dataset would enhance statistical confidence, deliverability with the 30°/15° combination is anticipated to rank between those of 30°/30° and 15°/15°.

Despite these limitations, we demonstrated the efficacy of dual arc increments in head and neck VMAT, with the 30°/15° combination being superior in terms of target dose coverage, doses to OARs, and plan delivery efficiency. Future research should explore applications to other treatment sites, particularly in scenarios where OARs are extremely close to the PTV, to obtain efficient VMAT plans.

## 5 CONCLUSION

We investigated the effects of dual arc increments on VMAT plan quality for head and neck cancer in terms of dose‐volumetric parameters for OARs and the PTV as well as plan complexity metrics. Substantial dose reductions to the spinal cord, brain stem, and parotid glands were achieved with comparable delivery efficiency and prescription dose coverages. Thus, we recommend dual arc increments combining 30° and 15° for each field in head and neck VMAT.

## AUTHOR CONTRIBUTIONS

Jin Hwa Choi performed the study analysis and wrote the manuscript. Hyejo Ryu and Do Hoon Oh performed patient selection, provided medical expertise, delineated and confirmed the PTVs, and analyzed the dose‐volumetric parameters. Lee Yoo collected the raw data, established the treatment plan, provided resources for study completion, and validated the study results. Minsoo Chun was responsible for the study design and execution, calculated the plan complexity metrics, performed the statistical analyses, and supervised the analysis and manuscript writing. All the authors reviewed the results and approved the final version of the manuscript.

## CONFLICT OF INTEREST STATEMENT

The authors declare no conflicts of interest.

## ETHICS STATEMENT

This research received ethical approval from the Institutional Review Board at Chung‐Ang University Gwang Myeong Hospital (Reference number: 2405‐164‐068).
